# Gene expression, purification, and functional characterization of recombinant conotoxin μ-TIIIA and TIIIAlaMut in *Escherichia coli* with clinical evaluation of antiwrinkle efficacy

**DOI:** 10.5114/bta/214377

**Published:** 2025-12-23

**Authors:** Diana Mikiewicz, Anna Mazurkiewicz-Pisarek, Magdalena Janczewska, Jolien De Waele, Alina Mazurkiewicz, Agata Stefanek, Frank Bosmans, Agnieszka Lew-Mirska, Przemysław Styczeń, Tomasz Ciach

**Affiliations:** 1 Science4Beauty LLC, Warsaw, Poland; 2 The Centre for Advanced Materials and Technologies, Warsaw University of Technology, Warsaw, Poland; 3 Molecular Physiology and Neurophysics Group, Department of Basic and Applied Medical Sciences, Faculty of Medicine and Health Sciences, University of Gent, Gent, Belgium; 4 Experimental Pharmacology, Department of Pharmaceutical Sciences, Faculty of Medicine and Pharmacy, Vrije Universiteit Brussel, Jette, Belgium; 5 Self Esteem Aesthetic Clinic LLC, Warsaw, Poland; 6 Aesthetic Medicine Clinic, Warsaw, Poland; 7 University of Technology, Faculty of Chemical and Process Engineering, Warsaw, Poland

**Keywords:** conotoxins, recombinant protein production, *Escherichia coli* expression system, patch-clamp method, antiaging activity

## Abstract

**Background:**

Conotoxins are small peptides known for their potent and selective activity on ion channels, offering potential applications in both medicine and cosmetology. This study aimed to design and validate recombinant conotoxin TIIIA and its mutant TIIIAlaMut, assess their biological activity on the voltage-gated Na^+^ (Nav) channel Nav1.4, and evaluate the antiwrinkle efficacy of a topical cream containing the recombinant peptide in a group of volunteers.

**Materials and methods:**

Fusion genes encoding *TRX::TIIIA* and *TRX::TIIIAlaMut* were cloned into the pDM vector and expressed in *Escherichia coli* S4B cells. The proteins were purified using Ni-NTA chromatography, cleaved with CNBr under optimized acidic conditions, and analyzed. Biological activity was assessed using two-electrode voltage-clamp electrophysiology in *Xenopus laevis* oocytes expressing the human Nav1.4 channel. Additionally, a conotoxin-containing cream was applied to 55 human volunteers in an application study assessing its antiaging effects.

**Results:**

Both recombinant genes were successfully expressed, purified, and activated. Electrophysiological measurements demonstrated their ability to inhibit Nav1.4 channel activity, including the version extracted directly from the cream. In the human study, 47% of participants reported a visible reduction in wrinkles. Additional benefits included evening of skin tone, reduced erythema, and balanced sebum production in oily skin types.

**Conclusion:**

This study describes the design, bacterial expression, and functional analysis of recombinant conotoxins TIIIA and TIIIAlaMut. Their bioactivity was confirmed on human Nav1.4 channels. The recombinant toxins, including the form extracted from the cream, showed effects comparable to a synthetic standard. Application tests demonstrated the conotoxin’s potential in cosmeceuticals, particularly in reducing periocular wrinkles and improving skin texture and tone.

## Introduction

Conotoxins are natural compounds found in the venom of cone snails from the *Conidae* family, which use them to immobilize and paralyze their prey (Terlau and Olivera 2004). These snails typically inhabit coral reefs and are predominantly found in tropical and subtropical waters, including the South China Sea, the Australian coast, and the Pacific Ocean. There are approximately 700 species of cone snails, all of which are venomous. Based on their dietary preferences, they can be categorized as worm hunters, mollusk hunters, or fish hunters (Olivera 1997; Duda et al. 2001).

Conotoxins are specialized peptides designed to target ion channels, receptors, and transporters in their prey, ensuring rapid immobilization. Their primary mode of action involves modulating or blocking ion channels, such as voltage-gated Na^+^ (Nav), K^+^ (Kv), and Ca^2+^ (Cav) channels, as well as ligand-gated ion channels like nicotinic acetylcholine receptors (Lewis et al. 2012). These peptides are structurally diverse and highly selective, having evolved to efficiently capture prey and defend against predators (Bergeron et al. 2013).

Due to their high specificity, conotoxins have become valuable tools for studying ion channels and hold potential therapeutic applications, particularly in targeting specific ion channels and glucose transporters. Ion channels are membrane proteins that regulate the movement of ions across the cell membrane and play crucial roles in both excitable and non-excitable cells, including neurons, muscle cells, renal tubules, and epithelial tissues.

Conotoxins are microproteins, typically under 40 amino acids in length, which facilitate their recombinant expression (Duque et al. 2019). They often form multiple disulfide bonds that stabilize their bioactive conformation, enhancing their potency, selectivity, and resistance to enzymatic degradation. A single cone snail’s venom may contain up to 100 different peptides, each serving a distinct function and collectively producing a potent effect on prey. Based on their molecular targets, conotoxins are classified into several types: ω-conotoxins block Cav channels to inhibit neurotransmitter release; α- and ψ-conotoxins block nicotinic acetylcholine receptors, causing neuromuscular blockade; μ- and δ-conotoxins target Nav channels in muscles; κ-conotoxins block Kv channels, increasing neuronal excitability; γ-conotoxins affect cation channels; and σ-conotoxins act as antagonists of serotonin 5HT3 receptors (Mir et al. 2016).

Due to their remarkable specificity, conotoxins are valuable biological tools for distinguishing closely related receptors, making significant contributions to neuroscience research. They have also demonstrated promise in pharmaceutical and cosmetic applications (Becker and Terlau 2008; Del Rio-Sancho et al. 2017; Pope and Deer 2013; Ramirez et al. 2017; Sun et al. 2019). Because many neurological and systemic disorders – such as epilepsy, schizophrenia, Tourette syndrome, Parkinson’s disease, and multiple sclerosis – are linked to malfunctioning ion channels, the small size, high potency, and selectivity of conotoxins position them as strong candidates for developing therapeutic and cosmetic solutions (Armishaw and Alewood 2005; Clark et al. 2010; Layer and McIntosh 2006; Miljanich 2004; Netirojjanakul and Miranda 2017).

For further research, we selected conotoxin TIIIA due to its myorelaxant properties, which result from the specific blockade of skeletal muscle Nav1.4 channels. This unique characteristic holds potential for application in cosmetology as part of daily antiwrinkle therapy.

In this study, we aimed to obtain active recombinant forms of the conotoxin TIIIA and its alanine-substituted mutant TIIIAlaMut using a bacterial expression system. These peptides were subsequently incorporated into the formulation of an anti-wrinkle cream intended to counteract skin aging processes at the molecular level.

## Materials and methods

### DNA manipulation, transformation, and sequencing

DNA restriction, ligation, and gel electrophoresis were performed using standard techniques (Sambrook et al. 1989). All bacterial transformations with plasmid DNA were carried out by electroporation using 1 mm cuvettes (BTX) and a MicroPulser™ electroporator (Bio-Rad, US). Electrocompetent *Escherichia coli* DH5α (New England Biolabs, UK Ltd.) and *E. coli* S4B cells (Mazurkiewicz-Pisarek et al. 2023; WO/2025/057018, this work) were prepared using standard procedures (Sambrook et al. 1989). The gene encoding conotoxin μ-TIIIA was synthesized by GenScript (Rijswijk, Netherlands).

All *E. coli* strains were propagated in LB broth (tryptone 10.0 g/l, yeast extract 5.0 g/l, NaCl 5.0 g/l, pH 7.2–7.5), supplemented with tetracycline (100 μg/ml). Plasmid DNA was isolated using the Plasmid Mini Isolation Kit (A&A Biotechnology, Poland) according to the manufacturer’s instructions. All restriction enzymes, ligase, and DNA ladders were purchased from New England Biolabs (UK Ltd.) and used according to the manufacturer’s instructions. A prestained protein molecular weight marker was purchased from GE Healthcare (UK). The correctness of DNA sequences was confirmed by sequencing (Genomed, Poland).

### Construction of TRX::TIIIA and TRX::TIIIAlaMut fusion genes

#### Construction of TRX::TIIIA fusion gene

To obtain a recombinant soluble conotoxin TIIIA protein, a genetic construct encoding the fusion protein TRX::TIIIA was designed. This construct included the gene encoding conotoxin TIIIA and the gene encoding the leader protein thioredoxin (TRX). The TRX protein provides reducing conditions that facilitate the correct folding of proteins containing disulfide bridges. It frequently enables recombinant proteins to be expressed in a soluble form, significantly simplifying the purification process.

The nucleotide sequence of the *TRX* thioredoxin gene was modified using site-directed mutagenesis to replace the amino acid methionine (M) at position 37 with lysine (K). This modification ensured appropriate protein fragments after cleavage with cyanogen bromide (CNBr), which specifically cleaves at methionine residues.

The nucleotide sequence of the *TRX::TIIIA* fusion gene was optimized for bacterial codon usage and ordered from GenScript (Rijswijk, Netherlands). Restriction sites (NdeI, XbaI) were added. To enable protein purification using a Ni-NTA chromatography column, a sequence encoding six histidines (6His) and a short linker consisting of serine-glycine-serine (SGS) was added to the 5′ end of the construct. The *TRX::TIIIA* fusion gene was inserted into the pDM expression vector (Mazurkiewicz-Pisarek et al. 2023; WO/2025/057018), digested with NdeI/XbaI. The nucleotide sequences of the cloned genes were verified.

#### Construction of TRX::TIIIAlaMut fusion gene

**Figure 1:**
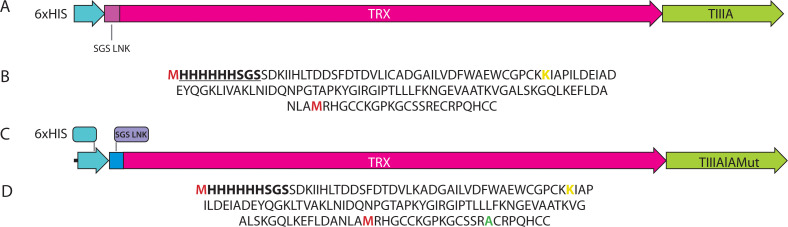
Schematic diagrams of (**A**) *TRX::TIIIA* and (**C**) *TRX::TIIIAlaMut* genetic constructs, and amino acid sequences of (**B**) TRX::TIIIA and (**D**) TRX::TIIIAlaMut. The amino acid methionine is marked in red, and the amino acid lysine is marked in yellow. The sequence including 6His and the SGS linker is shown in bold and underlined. The modified amino acid in TRX::TIIIAlaMut is highlighted in green

The recombinant fusion gene *TRX::TIIIAlaMut* was generated from the *TRX::TIIIA* gene using site-directed mutagenesis. A specific mutation was introduced to replace the glutamic acid (E) residue at position 15 with alanine (A). The nucleotide sequence of the modified gene was confirmed by sequencing. Schematic diagrams of the genetic constructs and amino acid sequences are presented in Figure 1.

### Construction of the E. coli expression strains

#### Construction of pDM/TRX::TIIIA plasmid

The expression vector pDM (a derivative of plasmid pBR322) was digested with NdeI/XbaI and ligated with a 428 bp NdeI/XbaI insert encoding the hybrid protein TRX::TIIIA. The codon usage of the hybrid gene was optimized for expression in *E. coli*. Transcription initiation in the constructed pDM/TRX+TIIIA plasmid is regulated by the *deoP1P2* promoter and includes a tetracycline resistance marker.

**Figure 2:**
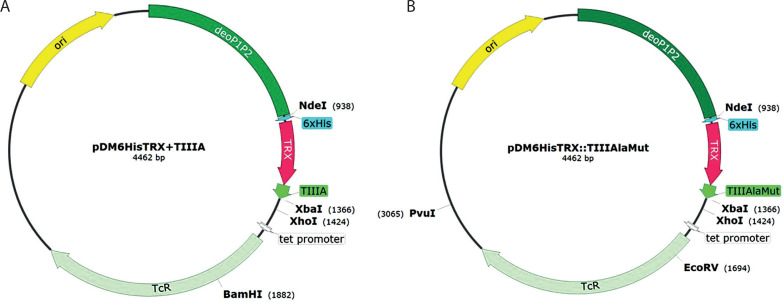
Construction scheme of the expression plasmids pDM/TRX+TIIIA and pDM/TRX+TIIIAlaMut. **A**) pDM/TRX+TIIIA contains the hybrid *TRX:TIIIA* gene under the *deoP1P2* promoter. **B**) pDM/TRX+TIIIAlaMut contains the hybrid *TRX:TIIIAlaMut* gene. Abbreviations: *TRX* – thioredoxin gene, *TIIIA, TIIIAlaMut* – conotoxin genes, tet promoter – tetracycline promoter, *TcR* – tetracycline resistance, ori – origin of replication

The final plasmid, named pDM/TRX+TIIIA, was used to transform the *E. coli* S4B strain developed in the Science4Beauty LLC laboratory. Figure 2 illustrates the strategy used to construct the pDM/TRX+TIIIA expression vector. A production strain of *E. coli* S4B containing the pDM/TRX+TIIIA construct – where the conotoxin *TIIIA* gene is fused to the thioredoxin leader protein gene, enabling the production of the target protein in a soluble form – was created.

Work was then carried out to optimize the culture conditions for the newly developed genetic construct and bacterial strain to achieve the highest possible expression of the recombinant conotoxin *TIIIA* gene.

#### Construction of pDM/TRX::TIIIAlaMut plasmid

The recombinant plasmid pDM/TRX::TIIIAlaMut was constructed using the previously prepared *TRX::TIIIAlaMut* fusion gene. The final plasmid retained all regulatory elements of the parent vector, including the *deoP1P2* promoter and the tetracycline resistance marker. The codon usage of the *TRX::TIIIAlaMut* gene was optimized for expression in *E. coli*. The verified construct was introduced into the production strain *E. coli* S4B, developed at Science4Beauty LLC, for recombinant expression of the soluble TRX-fused conotoxin mutant. The construction scheme of the final pDM/TRX::TIIIAlaMut expression vector is shown in Figure 2.

### Cell culture and purification of TRX+TIIIA and TRX::TIIIAlaMut fusion proteins

#### Purification of TRX+TIIIA fusion proteins

The prokaryotic expression vector pDM/TRX+TIIIA was introduced by electroporation into the *E. coli* S4B expression strain. On a laboratory scale, *E. coli* S4B strains were grown in LB broth supplemented with tetracycline (100 μg/ml) at 30°C, 150 rpm, for 18 h, reaching an OD_600_ of 3.2 to 3.5. Cultures were inoculated using stock material stored at –70°C (500 μl stock per 500 ml LB medium). Bacterial stocks were deposited in the strain bank collection at Science4Beauty LLC and prepared by mixing bacterial culture (OD_600_ ≈ 0.8) with 20% glycerol in a 1:1 ratio.

After 18 h of growth, the cultures were centrifuged (10 min at 8,000 rpm). The biomass from 1 l of culture was resuspended in 50 ml dissolution buffer (50 mM Tris-HCl, pH 7.8; 300 mM NaCl), sonicated (40% amplitude, 15 s on/5 s off, for 75 min, on ice), and centrifuged twice (15 min at 11,500 rpm). The clarified supernatant was applied to a Ni-NTA affinity column pre-equilibrated with calibration buffer.

The column was washed with wash buffer, and the TRX+TIIIA recombinant protein was eluted using elution buffer. The flow rate during sample loading was 1.0 ml/min; washing was performed at 1.5 ml/min; and elution was at 2.0 ml/min. Buffers used for protein purification – calibration buffer: 50 mM phosphate buffer (pH 7.8), 500 mM NaCl, 10 mM imidazole; Wash buffer: 50 mM phosphate buffer (pH 7.8), 500 mM NaCl, 20 mM imidazole; Elution buffer: 50 mM phosphate buffer (pH 7.8), 500 mM NaCl, 150 mM imidazole. Fractions were collected in 5 ml increments. Protein separation was performed on a Bio-Rad DuoFlow system using a chromatography column from the same manufacturer. After elution, 4 mM GSH (reduced glutathione) and 1 mM GSSG (glutathione disulfide, the oxidized form) were added to the collected fractions to ensure proper formation of disulfide bridges.

#### Purification of TRX+TIIIAlaMut fusion proteins

The expression plasmid pDM/TRX::TIIIAlaMut was introduced into the *E. coli* S4B strain by electroporation. Cultures were grown in LB medium supplemented with tetracycline (100 μg/ml) at 30°C and 150 rpm for 18 h, reaching an OD_600_ of 3.2–3.5. Inoculation was performed using 500 μl of frozen stock (OD_600_ ≈ 0.8, stored in 20% glycerol at –70°C) per 500 ml of medium. These strains are maintained at –70°C in the Science4Beauty LLC strain bank. After growth, cells from 1 l of culture were harvested at 8,000 rpm, resuspended in lysis buffer (50 mM Tris-HCl, pH 7.8; 300 mM NaCl), and sonicated under controlled conditions (40% amplitude, 15 s on and 5 s off, for 75 min, on ice). The lysate was centrifuged twice at 11,500 rpm for 15 min, and the resulting supernatant was applied to a Ni^2+^-affinity column (Bio-Rad DuoFlow System) equilibrated with binding buffer. Purification was performed using standard buffers: binding buffer (50 mM phosphate, 500 mM NaCl, 10 mM imidazole), wash buffer (same buffer with 20 mM imidazole), and elution buffer (with 150 mM imidazole). Elution was carried out at a flow rate of 2.0 ml/min, and fractions were collected every 5 ml. Following purification, 4 mM GSH and 1 mM GSSG were added to the eluted protein to facilitate correct disulfide bond formation.

### Cleavage of the TRX::TIIIA and TRX::TIIIAlaMut fusion proteins using CNBr

The purified recombinant fusion proteins were dialyzed for 48 h at 4°C against a buffer containing 50 mM Tris-HCl (pH 7.8) and 10% glycerol, using dialysis tubing with a molecular weight cutoff of 12–14 kDa. The dialysis buffer was replaced after 24 h. CNBr digestion was performed under acidic conditions, as the reagent specifically cleaves at methionine residues. CNBr reacts with the sulfur atom in the methionine side chain, resulting in cleavage of the peptide bond on the carboxyl side. Because methionine is among the least abundant amino acids in proteins, this method allows precise, targeted cleavage of the fusion proteins (Andreev et al. 2010). To separate the recombinant conotoxins from the thioredoxin (TRX) leader protein, cleavage was carried out using a 100:1 molar ratio of CNBr to methionine residues under acidic conditions (Gross and Witkop 1962; Inglis and Edman 1970). The TRX::TIIIA and TRX::TIIIAlaMut fusion proteins each contain two methionine residues, providing the specific cleavage sites required for this reaction. The amount of CNBr used in each reaction was calculated based on the molar mass of the individual fusion proteins. All procedures related to gene construction, expression strain development, and the production of active recombinant peptides are described in the corresponding patent application WO/2025/057018 (Mazurkiewicz-Pisarek et al. 2024).

### Electrophysiological testing on Nav1.4 channel genes expressed in Xenopus laevis oocytes

The activity of the recombinant conotoxins was assessed using two-electrode voltage-clamp electrophysiology on Nav1.4 ion channels expressed in *Xenopus laevis* oocytes (Leipold and Olivera 2018; McIntosh et al. 1999). The *Xenopus* oocyte expression system is well-suited for electrophysiological studies of voltage-gated ion channels due to its low background of endogenous channels and the large size of the oocytes (Dascal 1987). The human Nav1.4 (hNav1.4; NM_000334.4, OriGene Technologies, USA) gene was co-expressed with the human β1-subunit (NM_001037.5, GenScript, USA) at a 1:5 molar ratio by microinjecting capped RNA (cRNA) into defolliculated oocytes. Electrophysiological recordings were performed 1–2 days after injection. Oocytes were maintained at 17 °C in Barth’s medium (88 mM NaCl, 1 mM KCl, 5 mM HEPES, 2.4 mM NaHCO_3_, 0.41 mM CaCl_2_, 0.82 mM MgSO_4_, 0.33 mM Ca(NO_3_)_2_, and 50 μg/ml gentamycin, pH adjusted to 7.4 with NaOH).

Channel kinetics were examined using a two-electrode voltage-clamp setup (OC-725C, Warner Instruments, USA) with a 150 μl recording chamber. Data were filtered at 4 kHz and digitized at 20 kHz using pClamp10 software (Molecular Devices, USA). Micro-electrodes filled with 3 M KCl had resistances of 0.5–1 MΩ. The external ND100 recording solution contained 100 mM NaCl, 5 mM HEPES, 1 mM MgCl_2_, and 1.8 mM CaCl_2_ at pH 7.6 (adjusted with NaOH). All experiments were conducted at room temperature (~21°C). Leak and background conductances, identified by blocking Nav channels with tetrodotoxin (TTX), were subtracted from all current recordings. Voltage-activation relationships were obtained from peak currents, and conductance (G) was calculated and fit with a Boltzmann function using the equation: G/G_*max*_ = [1 + e^-zf^(V–V_1/2_)/RT]^-1^, where G/G_max_ is the normalized conductance, z is the equivalent charge, V_1/2_ is the half-activation voltage, F is Faraday’s constant, R is the gas constant, and T is the absolute temperature. Offline data analysis was performed using Clampfit10 (Molecular Devices, USA), Excel (Microsoft Office, USA), and Prism 8 (GraphPad, USA).

### Clinical efficacy testing

This application study was conducted between May 2023 and August 2023 and was approved by the Bioethics Committee at the District Medical Chamber in Warsaw in June (protocol code 1444/23 KB). The study was designed to assess the efficacy and user satisfaction of a novel cosmetic cream formulation containing the active ingredient conotoxin TIIIA. The cream was developed specifically for this research, and its complete composition is described in the corresponding patent application WO/2025/052257 (Janczewska et al. 2023). This *in vivo* study focused on evaluating both the performance and the user experience associated with the cream.

#### Treatment protocol

A total of 55 healthy adult volunteers (37 women and 18 men, aged 25–55 years; average age 40.6) were anticipated for recruitment. All participants provided written informed consent before enrolment. Each volunteer was pre-screened by a licensed physician to confirm eligibility based on the study’s inclusion and exclusion criteria.

#### Inclusion criteria

To qualify for participation in the study, the following inclusion criteria were applied: 1) age 25–55 years, 2) good general health, 3) written consent to participate in the study and acceptance of its procedures.

#### Exclusion criteria

To ensure the safety and accuracy of the clinical trial involving the cosmetic cream, it was essential to establish exclusion criteria. These criteria helped identify participants at risk of adverse reactions or whose involvement could compromise the reliability of the study results. The exclusion criteria for this study were as follows: 1) taking oral retinoids within the past 6 months, 2) skin and connective tissue diseases (e.g., systemic lupus erythematosus, collagenopathy, cutaneous porphyria), 3) active or frequently recurring Herpes simplex infection (cold sores), 4) use of medications that may affect skin condition (including tetracycline antibiotics, immunosuppressants such as cortisone and its derivatives, and anticoagulants such as dipyridamole and coumarin derivatives) within the past 6 months, 5) immunocompromised conditions (including active HIV infection), 6) pregnancy and breastfeeding, 7) uncontrolled hypertension, 8) unregulated diabetes, 9) vitiligo or disorders of melanin production (e.g., hypermelanosis), 10) tattoos in the treated areas, 11) constant use of anti-inflammatory medications, 12) history of allergic reactions to ingredients of the tested cosmetic formulations, 13) tendency to develop scarring, or having undergone aesthetic medicine or cosmetic surgery procedures within 4 weeks before or during the study.

All clinical assessments, surveys, and imaging procedures were conducted by trained personnel experienced in dermatological evaluation. Skin condition was assessed using high-resolution 3D imaging systems, specifically the VECTRA^®^ H2 (Canfield Scientific, USA) or FOTOMEDICUS (ELFO^®^, Poland), both of which enable quantitative analysis of wrinkles and skin tone.

Each study participant received two coded products labeled “A” and “B”. Participants were instructed to apply product A for 4 weeks, followed by product B for an additional 4 weeks. Product A served as the placebo formulation, while product B differed only in the presence of the active ingredient conotoxin TIIIA; all other excipients and formulation parameters were identical. Between the two application phases, an intermediate clinical assessment was performed, including high-resolution photographic documentation, 3D imaging using the VECTRA^®^ H2 (Canfield Scientific, USA) or FOTOMEDICUS (ELFO^®^, Poland) system, and a standardized user satisfaction questionnaire.

This within-subject design allowed each participant to serve as their own control, enabling a direct comparison of the placebo and active formulations while minimizing inter-individual variability. Participants remained blinded to the identity of each product throughout the study to reduce bias.

The cream was applied twice daily (morning and evening) to the facial skin. The application period lasted 4 weeks, with a progress questionnaire completed after 2 weeks. After 4 weeks, participants underwent clinical re-assessment and imaging. Primary outcomes included quantitative evaluation of skin texture and tone using 3D imaging and subjective assessments of product performance collected through structured questionnaires.

#### Methodology – image and data analysis

Image analysis was performed using specialized software capable of quantifying wrinkle depth through color-coded mapping, where red indicates deeper skin depressions and green represents shallower lesions.

Photographs taken before and after product application were analyzed by superimposing corresponding images and examining characteristic facial regions, including forehead wrinkles and periorbital lines.

Survey data were evaluated by the Principal Investigator based on anonymized participant responses to the complete set of questionnaire items.

The results were expressed as the percentage ratio of positive to negative responses, with answers marked as “hard to say” classified as neutral.

Throughout the study period, no adverse dermatological reactions or pharmacologically significant side effects were reported. Application sites remained free from irritation or inflammation, and no participants required medical treatment or withdrawal due to adverse effects.

## Results

### Designing of the pDM/TRX+TIIIA and pDM/TRX+TIIIAlaMut plasmids

The construction of the conotoxin expression plasmids pDM/TRX+TIIIA and pDM/TRX+TIIIAlaMut, based on the pDM vector, is illustrated in Figure 2. The DNA sequences encoding the *TRX::TIIIA* and *TRX+TIIIAlaMut* fusion genes include a modified thioredoxin fragment that enables the production of the recombinant protein in a soluble form. An *N*-terminal 6×His tag was incorporated to facilitate purification on a Ni-NTA agarose matrix. The presence and correctness of the inserts were confirmed by DNA sequencing.

### Protein production and analysis

The newly constructed plasmids pDM/TRX+TIIIA and pDM/TRX+TIIIAlaMut were introduced into *E. coli* S4B electrocompetent cells by electroporation, and intracellular expression was achieved. As expected, the expressed fusion proteins accumulated in the cytoplasm in a soluble form.

Following low-pressure liquid chromatography (LPLC) purification and 48-h dialysis, the TRX+TIIIA and TRX::TIIIAlaMut fusion proteins (1.0 mg/ml each) were subjected to CNBr cleavage. The reactions were carried out under acidic conditions at +4°C and at room temperature in the dark, with continuous stirring. A 100:1 molar excess of CNBr relative to methionine residues was used. For each milligram of fusion protein, 1.375 mg of CNBr was added, corresponding to 2.60 μl of a 5 M CNBr solution in acetonitrile (CH_3_CN; density 1.093 g/ml).

**Figure 3:**
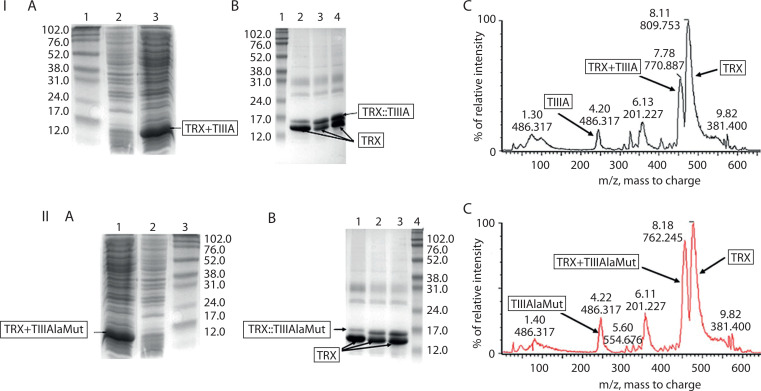
Expression and purification of TIIIA and TIIIAlaMut from *Escherichia coli*. **I**) TIIIA. **A**) SDS-PAGE analysis of TIIIA expression. Lane 1: molecular-weight marker; lane 2: total protein from *E. coli* S4B without insert; lane 3: total protein from *E. coli* S4B/pDM-TIIIA. **B**) SDS-PAGE analysis of CNBr-digested TRX::TIIIA under different conditions. Lane 1: molecular-weight marker; lanes 2–4: digestion in 0.3 M HCl, 0.2 M HCl, and 0.1 M HCl for 3 h, respectively. **C**) LC-MS chromatogram illustrating separation of the TRX::TIIIA fusion protein. **II**) TIIIAlaMut. **A**) SDS-PAGE analysis of TIIIAlaMut expression. Lane 1: molecular-weight marker; lane 2: total protein from *E. coli* S4B without insert; lane 3: total protein from *E. coli* S4B/pDM-TIIIAlaMut. **B**) SDS-PAGE analysis of CNBr-digested TRX::TIIIAlaMut under different conditions. Lane 1: molecular-weight marker; lanes 2–4: digestion in 0.3 M HCl, 0.2 M HCl, and 0.1 M HCl for 3 h, respectively. **C**) LC–MS chromatogram showing separation of the TRX::TIIIAlaMut fusion protein

The digested samples were analyzed by LC-MS (liquid chromatography-mass spectrometry). Based on these analyses, the optimal digestion conditions for TRX+TIIIA were determined to be 0.1 M HCl for 3 h at room temperature, in the dark, with continuous stirring. SDS-PAGE analysis could not detect the released conotoxins due to their low molecular weight (approximately 2.6 kDa), but the digestion pattern was confirmed by LC-MS. The corresponding SDS-PAGE gel images and LC-MS chromatograms are presented in Figure 3.

### Biological activity

Recombinant conotoxins TIIIA, TIIIAlaMut, conotoxin TIIIA extracted from the cream formulation, and the synthetic standard CnIIIC (Alomone Labs, Ltd., Israel) were tested on Nav1.4 channels expressed in *Xenopus laevis* oocytes at concentrations of 1 or 0.1 μM. Rapid and reproducible solution exchange (< 300 ms) was achieved using a 150 μl funnel-shaped oocyte chamber combined with a vertical solution flow delivered through a collector positioned next to the oocyte. Voltage-current relationships recorded from the same oocyte before and after administration of the tested compound were compared to assess channel inhibition.

**Figure 4:**
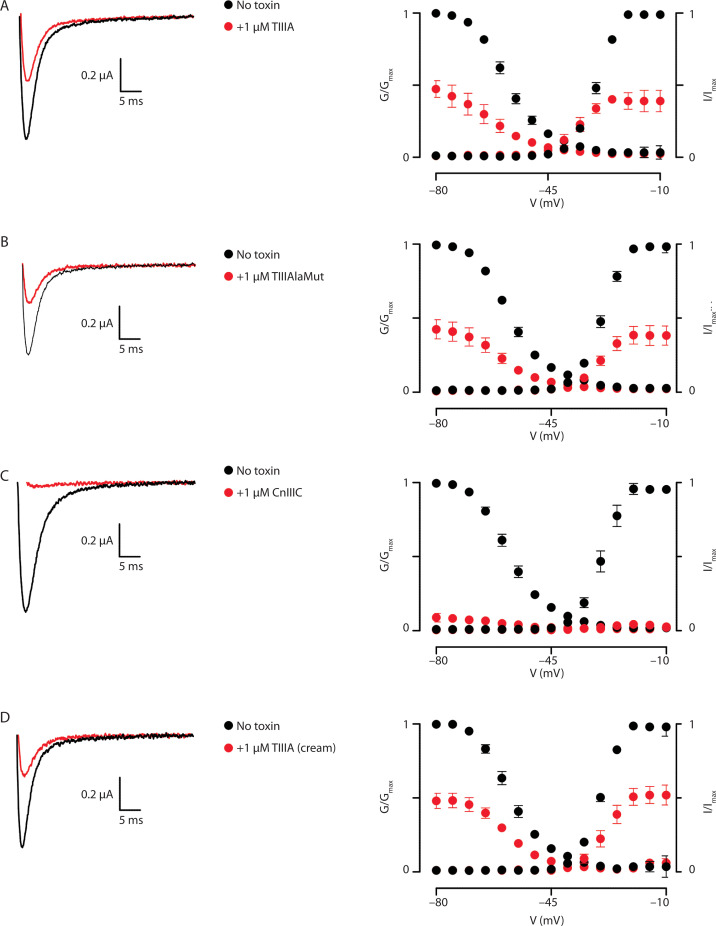
Biological characterization of conotoxin activity using *Xenopus laevis* oocytes expressing hNav1.4for: **A**) conotoxin TIIIA, **B**) conotoxin TIIIAlaMut, **C**) standard CnIIIC, **D**) conotoxin TIIIA extracted from cream. Left: representative hNav1.4 traces without and with toxin, demonstrating inhibitory effects. Right: effects on normalized conductance-voltage (G-V; open circles) and channel availability (I–V; filled circles) relationships. Error bars indicate SEM (*n* = 6 per condition)

The results of conotoxin activity on *X. laevis* oocytes expressing human Nav1.4 channels, recorded using the two-electrode voltage-clamp technique, are presented in Figure 4. Conotoxin TIIIA (Toxin A) exhibited an IC_50_ value of 9.7 μM, while the alanine-substituted conotoxin TIIIAlaMut (Toxin C) showed an IC_50_ of 3.8 μM. The synthetic standard CnIIIC (Toxin D) demonstrated an IC_50_ value of 33.6 nM. No full dose-response curve was obtained for conotoxin TIIIA extracted from the cream; however, it remained active on Nav1.4 channels at 0.1 μM.

### Efficacy test results

A total of 55 participants completed the full 4-week study period and were included in the final analysis. Results from the post-treatment questionnaires were analyzed descriptively, with data expressed as the percentage of positive responses for each evaluated aspect. Descriptive statistical methods were applied, and results are presented as percentage frequencies of positive responses. Given the observational design of the study, no inferential statistical tests were performed.

#### Overall satisfaction and user perception

**Figure 5:**
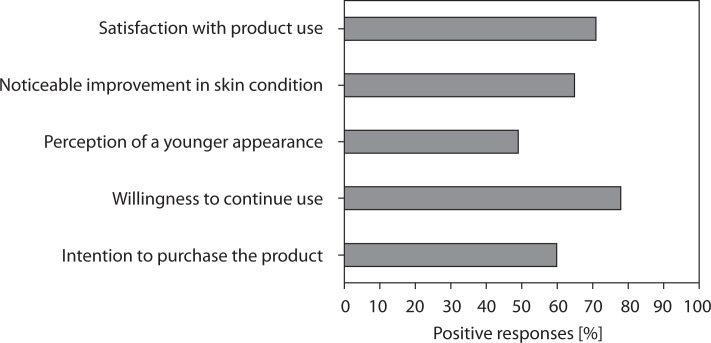
Results of the patient satisfaction survey: overall satisfaction and user perception

A graphical summary of the patient satisfaction survey, illustrating overall satisfaction and user perception, is presented in Figure 5. The results indicate a high level of user satisfaction: more than two-thirds of participants reported positive impressions, and nearly 80% expressed willingness to continue product use. Additionally, nearly half of the respondents perceived their skin as visibly younger, suggesting a moderate but noticeable aesthetic benefit.

#### Evaluation of product characteristics

**Figure 6:**
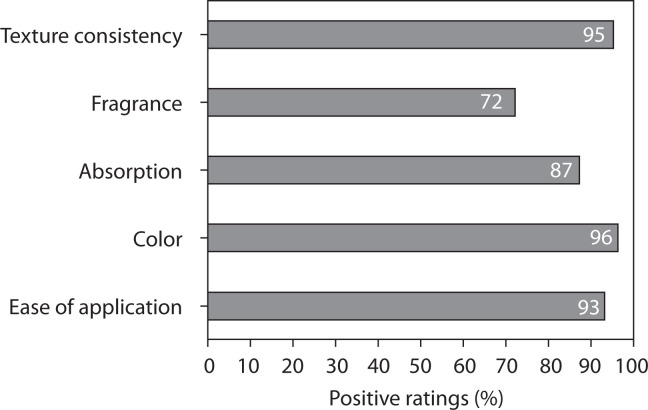
Patient satisfaction survey results for evaluation of product characteristics

The patient survey results evaluating product characteristics are shown in Figure 6. The cosmetic formulation was very well tolerated in terms of its physical and sensory properties. Over 90% of respondents rated texture, color, and application favorably, demonstrating excellent consumer acceptance.

#### Perceived improvement in skin condition

**Figure 7:**
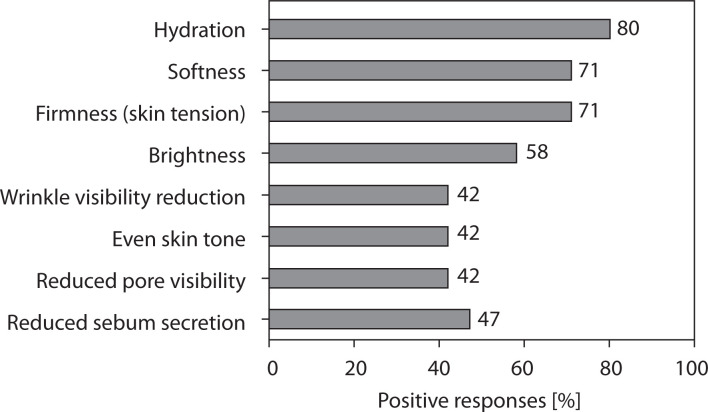
Graphical representation of patient-reported improvements in skin condition

The graphical representation of patient satisfaction survey results regarding perceived improvement in skin condition is shown in Figure 7. Most participants reported improvements in hydration, softness, and firmness, confirming the moisturizing and smoothing effects of the tested formulation. Moderate improvements were noted in skin brightness and pore size reduction. Wrinkle reduction and improvements in skin tone were reported by approximately 40–45% of respondents, suggesting the potential for progressive long-term benefits with continued use.

#### Perceived changes in wrinkle parameters

**Figure 8:**
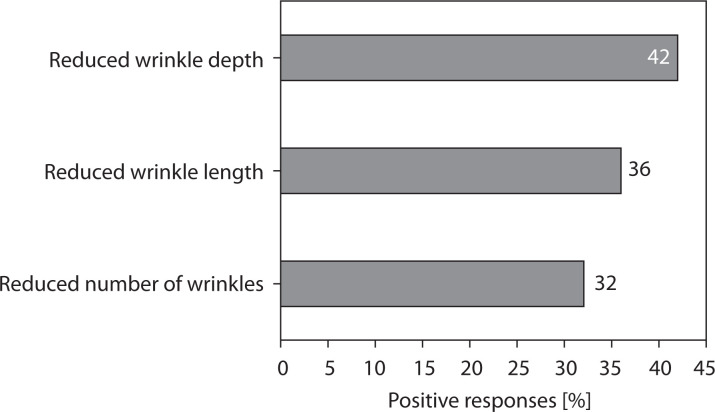
Patient satisfaction survey results regarding perceived changes in wrinkle parameters

The results of the patient satisfaction survey addressing perceived changes in wrinkle parameters are presented in Figure 8. Approximately one-third to two-fifths of participants perceived a visible reduction in wrinkle depth or prominence, which is consistent with the expected activity of the active ingredient, conotoxin TIIIA. These subjective findings complement the objective 3D imaging assessments, together indicating potential anti-aging effects of the formulation.

#### Overall outcomes

In the group of 55 participants who completed the study, a reduction was observed in both facial and static wrinkles, including “crow’s feet” and lower-eyelid wrinkles, as confirmed by the documented clinical cases. These regions are particularly challenging to treat with botulinum toxin, underscoring the effectiveness of the tested formulation. Among a subset of volunteers with upper-eyelid ptosis, a noticeable lifting effect was recorded. This outcome may be attributed to relaxation of the orbicularis oculi muscle, which lies very close to the skin and is nearly fused with it. Several participants who had previously struggled with this condition demonstrated clear improvement.

It is important to emphasize that the eye area is one of the most difficult regions to treat aesthetically due to its thinner epidermis, reduced subcutaneous tissue, and lower muscle mass compared to, for example, the forehead musculature. Despite these challenges, a significant improvement was also observed in static wrinkles in other facial areas, including the nasolabial folds and marionette lines. The most pronounced effect, however, was noted in the periocular region, with 47% of all volunteers reporting visible wrinkle reduction.

**Figure 9:**
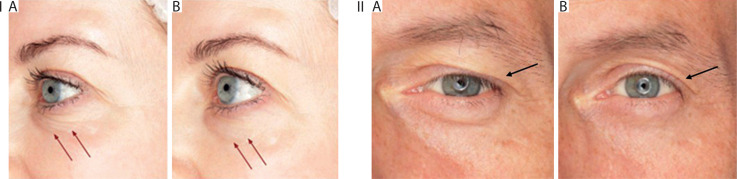
Representative before/after images of study participants. **I**) Visible reduction of periocular wrinkles, smoother skin texture, and softened crow’s feet. **II**) Reduction of upper-eyelid ptosis. **A)** Before treatment. **B)** After 28 days of using the cream containing conotoxin TIIIA

A cumulative effect of the cream was evident in a subset of participants who experienced enhanced skin texture and smoothness with continued use. Additional benefits included evening of skin tone and a reduction in erythema. These findings may suggest supplementary properties of the conotoxin ingredient, potentially analogous to botulinum toxin in its mechanism of modulating nerve-mediated vasodilation. Participants with oily or combination skin reported improvements in sebum control: 49% noted reduced skin shine, while 44% reported better regulation of sebum production. Representative images of the clinical outcomes are shown in Figure 9.

Importantly, the formulation demonstrated an excellent safety profile. No adverse dermatological reactions, irritation, or discomfort were reported during the four-week study period, confirming the good tolerance and suitability of the product for routine facial use.

## Discussion

In this study, we developed and characterized recombinant forms of conotoxin μ-TIIIA and its mutant TIIIAlaMut using a bacterial *E. coli* expression system. The use of a thioredoxin fusion enabled the production of soluble peptide forms, representing a significant improvement over earlier approaches in which incorrect folding of conotoxins in *E. coli* limited production yields (Becker and Terlau 2008; Klint et al. 2013). The recombinant peptides were efficiently cleaved from the fusion partner using CNBr, and the reaction conditions were optimized to minimize peptide degradation. Electrophysiological studies on *Xenopus laevis* oocytes expressing Nav1.4 channels demonstrated that both TIIIA and TIIIAlaMut retained their ability to inhibit Nav channel conductance, comparable to the synthetic CnIIIC standard, although with lower affinity. Similar findings have been reported for other μ-conotoxins, which exhibit high selectivity for Nav1.4 channels and show therapeutic potential in modulating skeletal muscle excitability (Green et al. 2014; Pei et al. 2024). Importantly, peptide activity was also confirmed after extraction from a completed cosmetic formulation, indicating structural stability and resistance to degradation during formulation and storage.

The 55-participant application study demonstrated a clear antiwrinkle effect, particularly in the periocular region, where 47% of participants reported visible improvement. These observations are consistent with the known myorelaxant properties of μ-conotoxins, which block sodium channels in muscle tissue, promoting muscle relaxation and subsequent smoothing of the overlying skin (Zou et al. 2024). The observed lifting effect of the upper eyelid may be clinically relevant, particularly for individuals with mild eyelid ptosis, where traditional botulinum toxin injections can be less effective or may carry a higher risk of adverse outcomes (Musharbash and Chakra 2024).

The results of the study demonstrated a high level of participant satisfaction and excellent usability of the tested cream formulation. The mean satisfaction level reached approximately 71%, and 78% of participants declared their willingness to continue using the product after completing the study. The cream also exhibited very good cosmetic properties, with an overall approval rate of 88.6% across all evaluated sensory parameters, including texture, spreadability, absorption, and pleasantness of application. Participants reported noticeable improvements in general skin condition, particularly in hydration and softness, which were rated positively by at least 70% of users. Subjective assessments showed visible wrinkle reduction in approximately 30–40% of participants, supporting the antiaging potential of the formulation.

Additional benefits – such as improved skin tone, reduced erythema, and sebum regulation in individuals with oily skin – suggest that conotoxins may exert effects beyond muscle relaxation. It is plausible that these peptides modulate neurogenic signalling within the skin’s microvasculature or sebaceous glands, a hypothesis previously proposed in the context of botulinum toxin type A (Rahman et al. 2024; Dayel et al. 2024). An important aspect of our findings is the potential for synergistic use of TIIIA conotoxin with botulinum toxin in aesthetic applications. Previous literature indicates that agents with similar mechanisms but different sites of action within the motor unit can prolong the duration of clinical benefits (Coleman and Carruthers 2006). This raises the possibility of developing cosmetic products that support or extend the effects of injectable treatments.

The main limitations of this study include the relatively small sample size and the short, 4-week application period. Further randomized clinical trials with control groups and longer follow-up are needed to assess the durability and long-term safety of the effects. Moreover, although the *in vitro* and *in vivo* results were consistent, a full understanding of the mechanism of action of conotoxins in the skin requires additional molecular studies, including analyses of their interactions with ion channels in keratinocytes and fibroblasts.

## Conclusions

In this study, we successfully demonstrated that the *E. coli* expression system can be effectively used for the production of recombinant μ-conotoxin TIIIA and its mutant TIIIAlaMut. The developed methodology provides a scalable and cost-efficient alternative to chemical synthesis, enabling access to functional μ-conotoxin variants. Importantly, the recombinant toxins retained their biological activity not only in electrophysiological assays but also after incorporation into cosmetic formulations, confirming their structural stability and compatibility with formulation processes. The application tests confirmed the potential of TIIIA conotoxin as a biologically active ingredient in cosmeceuticals.

Topical application of a cream containing conotoxin TIIIA resulted in visible antiaging benefits, most notably in the reduction of periocular wrinkles and improvements in overall skin smoothness and tone. Collectively, these findings indicate that recombinant μ-conotoxin TIIIA is a promising cosmeceutical ingredient with measurable antiwrinkle effects. Its properties suggest potential use both as a stand-alone cosmetic active and in combination with botulinum toxin to enhance or prolong aesthetic treatment outcomes.
